# Production of adeno-associated virus vectors for *in vitro* and *in vivo* applications

**DOI:** 10.1038/s41598-019-49624-w

**Published:** 2019-09-19

**Authors:** Toyokazu Kimura, Beatriz Ferran, Yuko Tsukahara, Qifan Shang, Suveer Desai, Alessandra Fedoce, David Richard Pimentel, Ivan Luptak, Takeshi Adachi, Yasuo Ido, Reiko Matsui, Markus Michael Bachschmid

**Affiliations:** 1 Vascular Biology Section, Whitaker Cardiovascular Institute, Boston University School of Medicine, Boston, USA; 2 Cardiology, Whitaker Cardiovascular Institute, Boston University School of Medicine, Boston, USA; 3 Cardiovascular Medicine, National Defense Medical College, Tokorozawa, Japan

**Keywords:** Biological techniques, Genetic transduction

## Abstract

Delivering and expressing a gene of interest in cells or living animals has become a pivotal technique in biomedical research and gene therapy. Among viral delivery systems, adeno-associated viruses (AAVs) are relatively safe and demonstrate high gene transfer efficiency, low immunogenicity, stable long-term expression, and selective tissue tropism. Combined with modern gene technologies, such as cell-specific promoters, the Cre/lox system, and genome editing, AAVs represent a practical, rapid, and economical alternative to conditional knockout and transgenic mouse models. However, major obstacles remain for widespread AAV utilization, such as impractical purification strategies and low viral quantities. Here, we report an improved protocol to produce serotype-independent purified AAVs economically. Using a helper-free AAV system, we purified AAVs from HEK293T cell lysates and medium by polyethylene glycol precipitation with subsequent aqueous two-phase partitioning. Furthermore, we then implemented an iodixanol gradient purification, which resulted in preparations with purities adequate for *in vivo* use. Of note, we achieved titers of 10^10^–10^11^ viral genome copies per µl with a typical production volume of up to 1 ml while requiring five times less than the usual number of HEK293T cells used in standard protocols. For proof of concept, we verified *in vivo* transduction via Western blot, qPCR, luminescence, and immunohistochemistry. AAVs coding for glutaredoxin-1 (Glrx) shRNA successfully inhibited Glrx expression by ~66% in the liver and skeletal muscle. Our study provides an improved protocol for a more economical and efficient purified AAV preparation.

## Introduction

Delivering a gene of interest to cells or animals has become an essential technique in biomedical research. In recent years, adeno-associated viruses (AAVs) have been used for many *in vitro* and *in vivo* applications due to their high transduction efficiency, safety, and extended stable gene expression. Furthermore, several recent clinical trials demonstrated the full potential of AAVs for human gene therapy^1–4^.

AAVs belong to the *Parvoviridae* family and are small non-enveloped viruses containing a linear single-stranded (ss) DNA^5^. Wild-type AAVs, as part of their lysogenic cycle, can integrate into the AAVS1 site of human chromosome 19 or rarely at random locations^6^. AAVs engineered for research or gene therapy, however, do not incorporate into the genome and instead form episomal concatemers in the host cell nucleus^7^. These head-to-tail circular concatemers remain intact in non-dividing cells but are lost during mitosis. Thus post-mitotic tissues, such as neurons and cardiomyocytes, may express transgenes over several months^8^.

AAVs are ideal for gene therapy due to their low immunogenicity, restricted generation of neutralizing antibodies, and replication defectiveness. AAV production requires cytopathogenic effects, which only occur after co-infection with a helper adenovirus or herpesvirus^8,9^. Helper viruses are difficult to remove and may induce undesired effects such as inflammation in the host. Current AAV expression systems avoid using helper viruses and include the plasmid pHelper instead^8^, containing essential genes such as E2A and E4. Human embryonic kidney cells (HEK) 293T cells, which express SV40 large T antigen, supply additional necessary proteins^9^. This AAV production system is referred to as ‘helper-free’ and consists of three different plasmids encoding essential viral and helper genes: pHelper, AAV trans-plasmid comprising AAV replication (*Rep*) and capsid (*Cap*) genes, and AAV cis-plasmid encoding the gene of interest, promoter, and inverse terminal repeats (ITRs)^1^.

Early work used the capsid and viral machinery derived from AAV serotype 2 (AAV2). AAV2 is still the basis for most AAV systems, but now, engineered capsids including DJ and DJ8 with tissue-specific tropisms or higher infectivity are available^10^. The DJ serotype also shows efficient transfection of many cultured cells, making DJ especially suitable for cell culture and *in vivo* applications. Furthermore, specific promoters, the Cre/flox system, and gene editing via CRISPR render AAVs cell-specific and allow novel approaches in gene therapy and animal research. Most protocols recommend AAV purification from lysates of producer cells, grown in large cell stacks or cell culture factories to obtain sufficient AAVs for animal experiments^11^. However, producer cells also release large quantities of AAV into the culture medium^12–16^, which often remains unused. Combining reported techniques, we optimized our protocol to obtain AAVs and purify the viral particles from producer cells and medium efficiently.

We tested several published protocols, most of which are labor-intensive, have low virus yields, and often result in contaminated virus preparations. We developed a revised protocol that is economical and efficient for the majority of laboratories with conventional equipment and reagents. For proof of concept, we evaluated the *in vivo* and *in vitro* efficacy of the viral particles by AAV-mediated short hairpin glutaredoxin-1 (*Glrx*) gene knockdown.

## Materials and Methods

### Reagents, materials and antibodies

HEK293T (ATCC® CRL3216™) human embryonic kidney and the mouse myoblast cell line C2C12 (ATCC® CRL1772™) were obtained from ATCC (Manassas, VA). Dulbecco’s modified Eagle medium (DMEM) and the penicillin/streptomycin cocktail were obtained from Gibco (Grand Island, NY) and the fetal bovine serum (FBS) was obtained from Atlanta Biologicals (Flowery Branch, GA). pAAV-DJ Rep-Cap plasmid (VPK-420-DJ), and pAAV-DJ/8 Rep-Cap plasmid (VPK-420-DJ-8) were purchased from Cell Biolabs (San Diego, CA). pHelper (Accession #: AF369965), pAAV-R2C6 (Accession #: AF369963), and pAAV-MCS (Accession #: AF396260) were obtained from Stratagene (CA, USA). The pCR8 vector was part of the Invitrogen TOPO cloning kit (K250020), and the Gateway system containing LR Clonase was from Invitrogen (Thermo Fisher Scientific, Waltham, MA).

Anion exchange columns and miniprep silica columns were from Epoch Life Science (Sugar Land, TX), silver nitrate solution and polyethyleneimine (PEI) were from Polysciences (Warrington, PA). Pluronic P68 and Optiprep^TM^ Axis-Shield (Dundee, Scotland) were ordered from Sigma-Aldrich (St. Louis, MO). Amicon Ultra-15 Centrifugal Filter Units (100,000 MWCO) were from Millipore (Burlington, MA). Type 70.1 Beckman rotor and Quick-Seal Centrifuge Tubes (Polypropylene, 13.5 ml, 16 × 76 mm) were obtained from Beckman Coulter (Brea, CA). Anti- green fluorescent protein (GFP) rabbit polyclonal antibody (PA1-980A), which also recognizes mVenus was obtained from Thermo Fisher Scientific (Waltham, MA). Anti-Glrx goat antibody (AGRX-03) was from IMCO (Solna, Stockholm). Polyvinylidene fluoride (PVDF) membrane for Western blot was obtained from GE Healthcare (Little Chalfont, Buckinghamshire). Precision Plus^TM^ Protein Prestained Protein Standards (10–250 kD) and acrylamide 37.5:1 solution were obtained from Bio-Rad (Hercules, CA). Western blots were developed using Kwik Quant peroxidase substrate and imager (Kindle Biosciences, Boston, MA). NEB Stable competent *E. coli* and BioLux Gaussia Luciferase Assay Kit were purchased from New England BioLabs (Ipswich, MA). All other supplies were bought from Thermo Fisher Scientific (Waltham, MA).

### Animals

Male C57BL/6J mice were obtained from The Jackson Laboratory (Sacramento, CA). Mice were maintained in the animal facility at Boston University Medical Campus on a 12-hour light-dark cycle and fed standard chow *ad libitum*. C57BL/6J mice were retro-orbitally injected with 100 µl of a viral preparation containing 5 × 10^11^ virus genomes (vg) encoding short hairpin Glrx (AAV2-DJ/8-shGlrx-mVenus) or hairpin Control (AAV2-DJ/8-shControl-mVenus) in saline (titers were measured with ITR primers). Different amounts of AAV expressing secreted Gaussia luciferase (AAV2-DJ/8-Gluc; 1.5 × 10^11^ vg/µl; injection volume 25 µl, 50 µl, and 100 µl; titer was measured with ITR primers) were administered via the tail vein. To locally transduce the skeletal muscle, 50 µl containing 4.4 × 10^12^ vg of AAV2-6-shGlrx-mVenus or AAV2-6-shControl-mVenus was administered via intramuscular (*IM*) injection (titers were measured with ITR primers). Mice were euthanized 2 to 6 weeks after virus administration. The protocol AN-15526 was approved by the Institutional Animal Care and Use Committee at Boston University School of Medicine.

### AAV cloning

The “Helper-free” AAV system comprised of pHelper, pAAV-MCS, pAAV-R2C6, was purchased from Stratagene (San Diego, CA) and capsid encoding plasmids pAAV-DJ and pAAV-DJ/8 were from Cell Biolabs (San Diego, CA). See ‘reagents, materials, and antibodies’ section and supplement for order details, accession number, plasmid maps, and sequence information. mVenus was inserted at the HindIII site of pAAV-MCS. A Gateway cassette (attB1 and attB2) was inserted at MluI site to facilitate the insertion of the U6 promoter expression cassette. Control shRNA (target sequence: ACACCTATACAACGGTA) and mouse Glrx short hairpin RNA (shRNA; target sequence: AGTCCACTTTCTAAAGAA) were cloned as described previously^17^, and a CMV enhancer sequence was added upstream of the U6 promoter to enhance shRNA expression^18^. The shRNA expression cassette was designed according to the guidelines published by Miyagishi *et al*.^19^ introducing a 17 nucleotide (nt) counting double strand stem with a 21 nt long loop (GCTGCGTTCAAGAGATGCGGT). The shRNA construct was created by tandem PCR with human U6 promoter. These sequences were inserted in the pCR8 vector, and the AAV plasmid expressing shRNA and mVenus was created by LR reaction according to the manufacturer’s instructions (please find sequence maps in the supplement). AAVs were produced by co-transfection of pHelper, pAAV ITR-expression vector, and pAAV *Rep*-*Cap* genes in a 1:1:1 molar ratio normalized to the plasmid size (see supplement).

Plasmids were prepared by alkaline lysis followed by anion-exchange chromatography (Epoch Life Science, Sugar Land, TX) and isopropanol glass filter-assisted precipitation. A low-cost plasmid preparation protocol is provided as Supplementary Information. The integrity of isolated plasmids was verified by restriction enzyme digestion with PvuII, ApaLI and ApaI and agarose gel electrophoresis. The identity of all constructs was confirmed by sequencing using the Genewiz (Boston, MA) services.

### AAV production and purification

The detailed protocol for AAV purification is provided as Supplementary Information. Briefly, 50–70% confluent HEK293T cells grown in DMEM supplemented with 5% FBS were triple transfected with pHelper, pAAV ITR-expression, and pAAV Rep-Cap plasmids using acidified PEI^20^ in five T150 flasks. After transfection, two medium changes to DMEM with FBS were performed. At day 5 post-transfection, media and cells were collected and processed separately. Cells were lysed in an acidic buffer, the homogenates were cleared from debris by centrifugation, and the pH was neutralized using HEPES buffer. AAVs were precipitated from lysates and medium with polyethylene glycol (PEG) 8000. The PEG-precipitated AAV was collected by centrifugation, followed by chloroform and an aqueous two-phase extraction in ammonium sulfate and PEG 8000^21,22^. For *in vivo* use, AAVs were further purified using discontinuous iodixanol gradient ultracentrifugation^23,24^. Three different pAAV Rep-Cap plasmids were used: pAAV-DJ (VPK-420-DJ, Cell Biolabs), to obtain AAV2-DJ particles for the *in vitro* studies; pAAV-DJ/8 (VPK-420-DJ-8, Cell Biolabs), to address the *in vivo* transduction to the liver by AAV2-DJ/8 particles; and pAAV-R2C6 (pAAV-RC AF369963, Stratagene), to obtain virions with AAV2-6 serotype and address the transduction to the skeletal muscle.

### AAV titration and purity assessment

Primers binding within the AAV2 ITRs^25^ were used to measure the virus titer with quantitative polymerase chain reaction (qPCR). Before releasing the viral DNA from the particles, all extra-viral DNA was removed by digestion with DNase I. Then, the viral DNA was released by alkaline lysis. The qPCR was performed using the PowerUp™ SYBR™ Green Master Mix (Applied Biosystems, Foster City, CA), and primers against the ITRs and a primer pair that amplifies the short hairpin cassette (Forward: GTGGAAAGGACGAAACACCG; Reverse: GCTCCAAGGATCATCAACCAC) obtained from Invitrogen (Carlsbad, CA). The extracted viral DNA and a serial dilution of a viral plasmid containing ITRs and the short hairpin cassette as a standard were measured using the CFX96 Touch Real-Time PCR Detection System and the CFX Maestro Software (Bio-Rad, Hercules, CA). For details about AAV extraction and analysis, please refer to the Supplementary Information.

AAV capsid proteins were separated by sodium dodecyl sulfate-polyacrylamide gel electrophoresis (SDS-PAGE) and detected with fast silver staining to assess the purity of viral preparations^26–28^. Serial dilutions of bovine serum albumin (BSA; Sigma-Aldrich, St. Louis, MO) were used as protein standards. Images were obtained using an Epson scanner (Epson Perfection V800 Photo, Digital ICE Technologies). A detailed protocol is provided as Supplementary Information.

### Cell culture

Experiments were performed on primary hepatocytes from C57BL/6J mice and the C2C12 cell line. Cells were cultured in 12-well plates in high glucose DMEM containing 10% FBS and penicillin/streptomycin. C2C12 cells were made quiescent by reducing FBS to 1% in the medium. Cells were transduced with AAV containing either short hairpin Glrx (AAV2-DJ-shGlrx-mVenus) or short hairpin Control (AAV2-DJ-shControl-mVenus) at 1.56 × 10^6^ vg per well (titers were measured with ITR primers). Six days after infection, cells were harvested for further analysis of RNA and protein levels. Glucose concentrations were measured using a Contour Next One blood glucose meter and a single use glucose test strip (Bayer, Ascensia Diabetes Care, Parsippany, NJ) with 1 µl of cell culture medium. Acidification was measured with a Mettler Toledo (Columbus, OH) LE422 micro pH electrode.

### Luciferase activity assay

Four groups of four male mice were injected with either phosphate buffered saline (PBS) or AAV to overexpress the *Gaussia* Luciferase, as previously described in the “Animals” section. Blood was collected from the tail vein at 1–3 week intervals after AAV injection. Serum was separated from red cells by centrifugation at 1,000 × g at 4 °C for 10 minutes. Luciferase activity was measured in the serum with a BioLux *Gaussia* Luciferase Assay Kit according to the manufacturer’s protocol using a TD-20e Luminometer (Turner BioSystems, Sunnyvale, CA).

### Western blotting

Tissues or cell monolayers were homogenized in lysis buffer composed of 50 mM Tris pH 7.4, 150 mM NaCl, 5 mM EDTA, 1% NP-40, and supplemented with cOmplete^TM^ Mini Protease Inhibitor Cocktail (Roche Applied Science, Penzberg, Germany). After removing the debris by centrifugation, the protein concentration of lysates was measured with the DC^TM^ Protein Assay (Bio-Rad, Hercules, CA). Protein samples were prepared under reducing conditions, and 50 μg of total protein per lane were loaded on a NuPAGE 4–12% Bis-Tris gel (Invitrogen, Carlsbad, CA). After transferring proteins to a PVDF membrane using a Trans-Blot^TM^ Turbo Transfer System (Bio-Rad, Hercules, CA) and blocking with 5% non-fat dry milk powder (NFDM), the blots were incubated overnight at 4 °C with anti-GFP, anti-Glrx, and anti-β-tubulin antibodies, all diluted 2000-fold in 5% BSA. After incubating the blots for 1 hour with corresponding HRP-conjugated antibodies (diluted 5000-fold in 5% NFDM), the chemiluminescent signal was detected using the Hi/Lo Digital-ECL^TM^ Western Blot Detection Kit and the KwikQuant^TM^ Imager. The bands of interest were quantified using the ImageJ software.

### Reverse transcription (RT) and the quantitative polymerase chain reaction

Total RNA was extracted from cells or tissues with TRIzol reagent (Invitrogen, Carlsbad, CA) and the Direct-zol RNA MiniPrep Plus kit (Zymo Research, Irvine, CA). cDNAs were synthesized from 1 μg of total RNA using the High-Capacity RNA-to-cDNA™ kit (Applied Biosystems, Foster City, CA) according to the manufacturer’s instructions. Quantitative PCR was performed using TaqMan primers (Applied Biosystems, Foster City, CA) Glrx (Mm00728386_s1) and Actb (Mm01205647_g1) using CFX96 Touch Real-Time PCR Detection System and CFX Maestro Software (Bio-Rad, Hercules, CA). Expression changes were calculated by the comparative Ct (ΔΔCt) method.

### Immunofluorescence

Liver sections (10 μm) were cut with a Leica CM1950 Clinical Cryostat (Leica, Wetzlar, Germany), washed with PBS three times (3–5 minutes each time), fixed with 4% paraformaldehyde (PFA) in PBS for 10 minutes, and rinsed in PBS three times at room temperature. Samples were then blocked in 5% NFDM in PBST for 1 hour at room temperature. Rabbit anti-GFP (1:200, PA1-980A; ThermoScientific Pierce Products) was used as primary antibody in 5% NFDM in PBST and samples were incubated overnight at 4 °C. Sections were washed three times in PBS and incubated with the secondary antibody Alexa Fluor 594 (1:200, R37117; Thermo Scientific Pierce Products) and Hoechst 33342 (1:2000, H3570; Bioprobes) for 1 hour at room temperature. After incubation, slides were washed three times and mounted using FluorSave Reagent (Calbiochem). Individual images were acquired using the HS All-in-one Fluorescence Microscope BZ-9000E (Keyence, Osaka, Japan) with a 20x objective and the BZ-II Analyzer (Keyence, Osaka, Japan).

### Statistical analysis

Statistical analysis was performed using Prism 5.0 (GraphPad Software, La Jolla, CA). Means were compared between two groups by student’s t-test and two-tailed Mann-Whitney tests. More than two groups were analyzed with the non-parametric Kruskal-Wallis test followed by Dunn’s multiple comparison post-test. P values < 0.05 were considered statistically significant. The error was reported as the standard error of the mean (SEM).

### Regulatory

All experimental protocols were approved by Boston University Institutional Animal Care & Use Committee (IACUC) under animal protocol AN-15526 and Boston University Institutional Biosafety Committee (IBC) under protocol 16-983. All methods were carried out in accordance with the relevant and approved guidelines and regulations.

## Results

### Serotype-independent purification of AAVs

**Figure 1:**
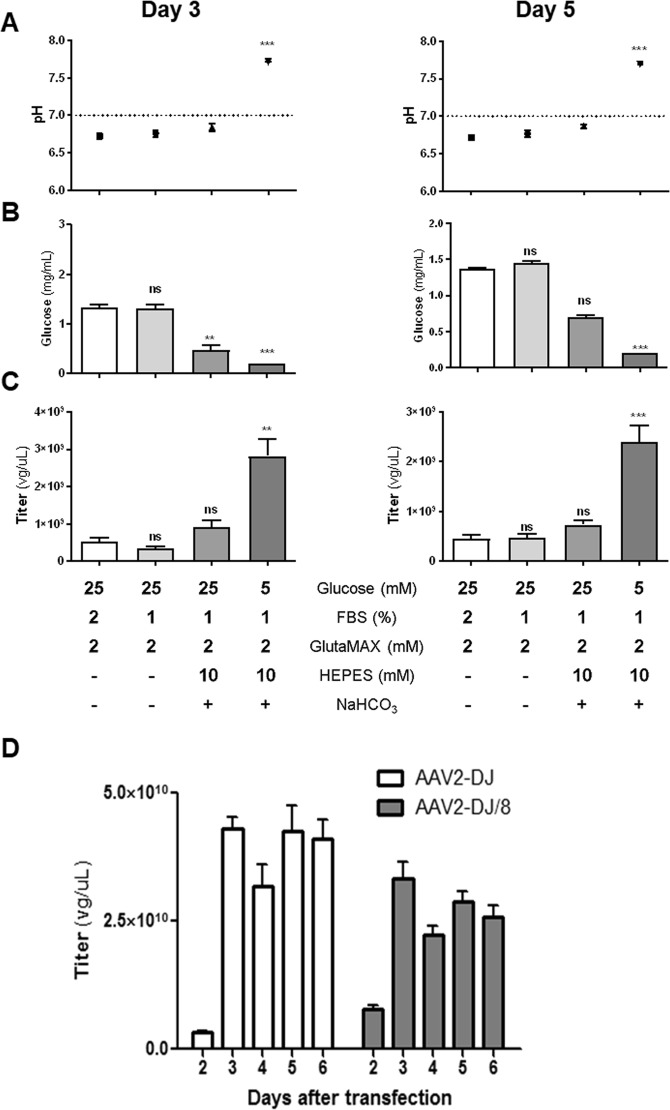
Comparison of AAV production using different media formulations and time points. The graph represents (**A**) pH, (**B**) glucose concentration, and (**C**) viral titers of AAV2-DJ-shGlrx-mVenus in the cell culture medium of HEK293T cells at days 3 and 5 post-transfection with viral plasmids. HEK293T cells were cultured for AAV production in supplemented DMEM as described in the table below. Statistical analysis was performed using the non-parametric Kruskal-Wallis test followed by Dunn’s multiple comparisons test. Statistical differences (n = 8; ns = not significant; **P < 0.01; ***P < 0.001; SEM) are compared with medium containing 2% FBS as a control. (**D**) The graph shows titers of the released AAV2-DJ-shGlrx-mVenus and AAV2-DJ/8-shGlrx-mVenus into the cell culture medium at different days post-transfection. The medium was composed of DMEM, 1% FBS, 1x Glutamax, 10 mM HEPES and addition of 0.075% sodium bicarbonate. On day 3, the medium was changed (n = 8; SEM).

Depending on the AAV serotype, HEK293T cells release a significant amount of the virus into the medium without cytopathic effects^12–15^. AAV2- and DJ-capsid based viruses, however, may remain bound to the cell surface via an intact heparin binding domain^12^. Reports have shown that serum reduction and mildly alkaline pH increase AAV production^29^. We tested several culture conditions to determine whether cells in an optimal or stress environment can promote viral production. Standard Dulbecco Modified Eagle Medium (DMEM) to grow HEK293T cells contains 25 mM of glucose and glutamine as a carbon and energy source as well as fetal bovine serum to sustain cell growth. Since glutamine spontaneously decomposes to form ammonia during storage and long-term culture, we employed the alternative GlutaMAX (L-alanyl-L-glutamine dipeptide). GlutaMAX can minimize ammonia build-up and media exhaustion during culture^30^. Due to protein contamination from fetal bovine serum after iodixanol purification (mainly albumin; protein band at 66 kDa), we initially reduced the fetal bovine serum to 1%. The reduced serum quantities minimally impacted the viral titer on either day 3 or 5 (Fig. 1A–C).

Upon viral protein expression, marked media acidification occurred on day 3 and 5 in standard 25 mM glucose (4.6 g/l) containing DMEM. The initial pH dropped from 8.0 to below 7.0 (Fig. 1A). To increase the buffering capacity of the 25 mM glucose DMEM and delay acidification, we supplemented the medium with cell culture compatible amounts of sodium bicarbonate and HEPES. Even in the presence of high amounts of the buffering compounds, the pH still dropped below 7.0 on day 3 and 5 with only a mild increase in viral quantity (Fig. 1C). As the releases of lactate from glycolysis is a common cause of cell culture medium acidification, we speculated that limiting glucose may prevent media acidification and increase viral particles. For further determination of the effect of glucose, we cultured AAV-producing cells in 5 mM glucose (1 g/l) DMEM with the same amounts of sodium bicarbonate and HEPES buffering, FBS, and stable glutamine. The pH stabilized at ~7.4 and glucose concentration dropped below the detection limit but the viral production increased ~3-fold. These data suggest that limiting glycolysis to stabilize the pH appears beneficial for viral production. Presumably, limiting glycolysis forced cultured producer cells to derive energy and biosynthetic building blocks through glutaminolysis^31^, a well-established effect observed in cultured cells^32,33^, immune cells^34^, and pluripotent stem cells^35^.

Overall, HEK293T cells showed robust AAV production under varying conditions, but in our hands, low glucose DMEM medium supplemented with 1% FBS, 1x Glutamax, 10 mM HEPES and addition of 0.075% sodium bicarbonate delivered the best results. Using this production medium and following the timeline described in the protocol, we quantified AAV2-DJ and AAV2-DJ/8 particles released at 2, 3, 4, 5 and 6 days after transfection (Fig. 1D). Producer cells released considerable amounts of viral particles at day 3, and after changing the medium, the titer increased again until day 5 to a steady level.

**Figure 2:**
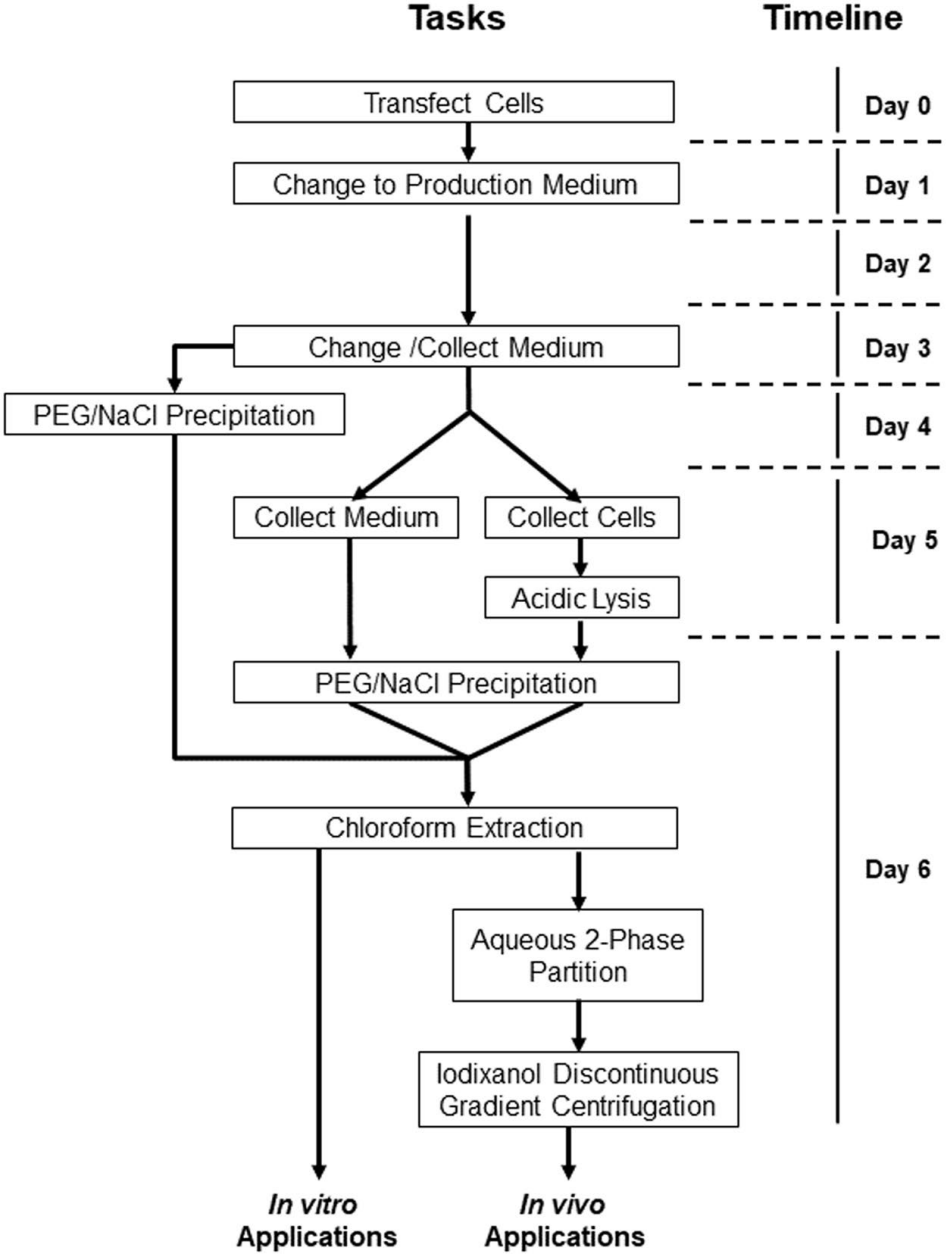
Flowchart of AAV purification. The helper-free AAV system comprises three types of plasmids (ITR-containing plasmid, AAV Rep-Cap plasmid, pHelper). Triple plasmid co-transfection with PEI into HEK293T cells was performed in five T150 flasks (day 0). On day 3 and day 5, 150 ml of medium were collected and subjected to AAV purification. On day 5, cells were detached with 0.5 M EDTA (pH 8.0), collected, and subjected to lysis in citrate buffer for further purification. AAVs were isolated from both cell lysate and medium, and the purification took 2 days, from day 5 to day 6. Cell lysate and medium were purified in the same manner after the PEG/NaCl precipitation. After aqueous two-phase partitioning, an iodixanol discontinuous gradient purification step was performed.

We tested several published protocols to purify AAVs. Most protocols are labor intensive, have low virus yields, and often result in contaminated virus preparations. Thus, we have developed a revised protocol as outlined in Fig. 2. AAVs can be concentrated and purified from cell culture medium by cost-effective PEG precipitation. The procedure includes two time points for medium collection on day 3 and 5 to increase virus yields. The delayed medium collection has negligible effects on AAV activity, which was also reported by others^12,14,15^.

HEK293T cells contain AAVs, and most protocols use harsh lysis conditions resulting in increased contamination with cellular proteins and DNA. These lysates require further extensive processing with DNase I/Benzonase^TM^ ^36,37^. In contrast, lysis of HEK293T producer cells in an acidic citrate buffer promotes AAV release with less contamination^38^. Furthermore, citrate complexes bivalent ions and the low pH may activate an intrinsic protease activity of viral capsid proteins^39^, likely aiding with the release of AAVs. We noted that these cell lysates prepared in acidic citrate buffer (110 mM citrate, pH 4.2) were markedly cleaner than conventional cell lysis, and subsequent purification steps removed any residual contaminants. Prior methods have focused on mainly using cell lysates to collect viruses. We confirmed that production medium contained significant amounts of AVVs in the range of 10^8^ to 10^10^ viral genomes per microliter (vg/µl), which allowed us to obtain final titers of 10^10^ to 10^11^ vg/µl of purified AAVs measured with qPCR using specific primers for the short hairpin region. However, using primers that amplify the ITR sequence, we obtained titers up to 10 times higher (in the range of 10^11^–10^12^ vg/μl). Therefore, the ITR sequence is universal but may overestimate the virus titer. Additional pilot experiments should always be performed to confirm the biological activity of the AAV^40–43^.

Using the AAV-containing media, we performed PEG precipitation, followed by lipid extraction with chloroform and aqueous two-phase partitioning with 10% (w/w) PEG and 13.2% (w/w) ammonium sulfate^21^. This sequence of purification steps allowed for most protein contaminants to precipitate or partition into the inter- and top PEG phases. AAVs remained soluble in the ammonium sulfate phase.

**Figure 3:**
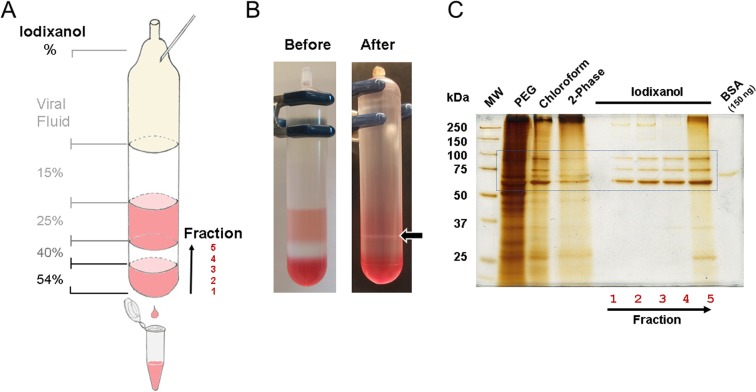
AAV iodixanol discontinuous gradient purification and purity assessment. (**A**) Serotype-independent purification of AAVs using a discontinuous iodixanol gradient and ultracentrifugation. The 15% iodixanol layer was underlayered with denser iodixanol solutions to create the step gradient. The 25% and 54% iodixanol layers included phenol red for better visualization. Complete AAVs concentrated in the 40% layer. For elution of the iodixanol gradient, a small hole was drilled into the bottom of the centrifuge tube using a 25 G needle and another 25 G needle was inserted at the top. Fractions of 1 ml for the 54% layer, three of 400 μl for the 40% layer, and 1 ml for the 25% interface were collected. (**B**) Actual iodixanol step gradient before and after centrifugation. Visible interphase (arrow) between the 40% and 25% layers consists of empty and incomplete AAV2-DJ/8 particles, which may contain fragments of viral ssDNA. (**C**) Silver-stained protein gel of AAV2-DJ/8 samples at different stages of purification. Box denotes the area of the viral capsid proteins VP1 (87 kDa), VP2 (73 kDa), and VP3 (62 kDa). The purification procedure, using PEG precipitation, chloroform extraction, and aqueous two-phase extraction gradually separated contaminating proteins. The final discontinuous iodixanol gradient ultracentrifugation yielded highly purified AAV particles in fractions 2–4. The viral genome copy number measured with qPCR was high in fractions 2–4 while fraction 5 contained ~10 times lower copy numbers, likely due to viral particles containing ssDNA fragments carried over from the 40% layer.

For *in vivo* use, we removed the remaining contaminants with a discontinuous iodixanol (OptiPrep^TM^) gradient ultracentrifugation^23,24^ using four layers of different iodixanol concentrations of 15, 25, 40, and 54% (Fig. 3A,B). The iodixanol gradient is also a useful step to remove empty or incomplete viral particles^14,44–46^. The isotonic and relatively inert nature of iodixanol maintains the AAVs potency. As high amounts of iodixanol may also cause kidney toxicity in already health-compromised animals^47–49^, we reduced the iodixanol concentration of the final virus suspension using centrifugal filter units.

To assess the purity of AAV preparations, we performed silver staining (Fig. 3C). The chloroform extraction step (lane 3) resulted in sufficiently pure AAV preparation suited for cell culture use. Fractions 2–4 collected from the 40% layer (lanes 6–8) showed three major bands corresponding to the AAV capsid proteins—virion proteins 1 (VP1; 87 kDa), 2 (VP2; 73 kDa), and 3 (VP3; 62 kDa)—with a purity greater than 90% and suitable for *in vivo* use. Samples above fraction 4 showed a band at ~66 kDa similar to the BSA standard, which likely represents medium-derived albumin and several other protein contaminants. Thus, iodixanol discontinuous gradient ultracentrifugation effectively removes most contaminants and results in highly purified and enriched fractions of active AAV.

### Testing AAVs *in vitro*

We produced AAV2-DJ and AAV2-DJ/8 with a bicistronic expression system coding for *Glrx* shRNA and mVenus, a variant of the yellow fluorescent protein, using the purification protocol without discontinuous iodixanol gradient.

We have previously reported that Glrx-deficient mice develop obesity and non-alcoholic fatty liver (NAFL) disease^50^. On the other hand, Glrx deficiency promotes angiogenesis in ischemic limbs in this mouse strain^51^. Glrx is a thiol transferase which removes GSH adducts from proteins^52^. GSH adducts are generated through oxidative post-translational modifications, especially at cysteine residues^53^, and regulate the function of transcription factors^51,54^, cytoskeletal assembly^55^, and signaling molecules^56,57^. Since the role of Glrx differs between tissues, there is the need to locally inhibit Glrx expression or manipulate Glrx expression in a tissue-specific manner.

AAV2-DJ has a hybrid capsid generated by DNA shuffling from different native serotypes^10^. These AAV vectors are considered to have high infectivity to tissues and cells compared to other native serotypes.

**Figure 4:**
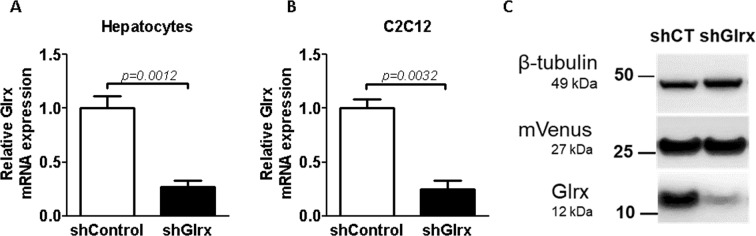
mRNA and protein levels in primary hepatocytes and C2C12 cells infected with AAV2-DJ-shGlrx-Venus. Glrx mRNA levels in primary hepatocytes (**A**), and C2C12 cells (**B**) after 6 and 4 days post infection, respectively, with either AAV2-DJ-shControl-mVenus (shControl) or AAV2-DJ-shGlrx-mVenus (shGlrx). Glrx mRNA expression was normalized to Actb mRNA, and the fold-change was compared to shControl (n = 6–7; SD). (**C**) Glrx and Venus protein levels in C2C12 cells from (**B**). β-tubulin served as a loading control. shCT: C2C12 cells infected with AAV2-DJ-shControl-mVenus.

Primary mouse hepatocytes in culture transduced with AAV2-DJ-shGlrx-mVenus exhibited a ~70% knockdown of Glrx expression compared to AAV2-DJ-shControl-mVenus infected cells (Fig. 4A). Quiescent confluent C2C12 cells showed similar results for mRNA expression and protein levels after 4 days (Fig. 4B,C). AAV2-DJ/8 was unable to transduce cultured cells (data not shown). AAV-delivered shRNA is much more potent compared to the application of siRNA, with which we have experienced difficulties in silencing *Glrx* gene expression in differentiated C2C12 cells.

### Testing AAVs *in vivo*

**Figure 5:**
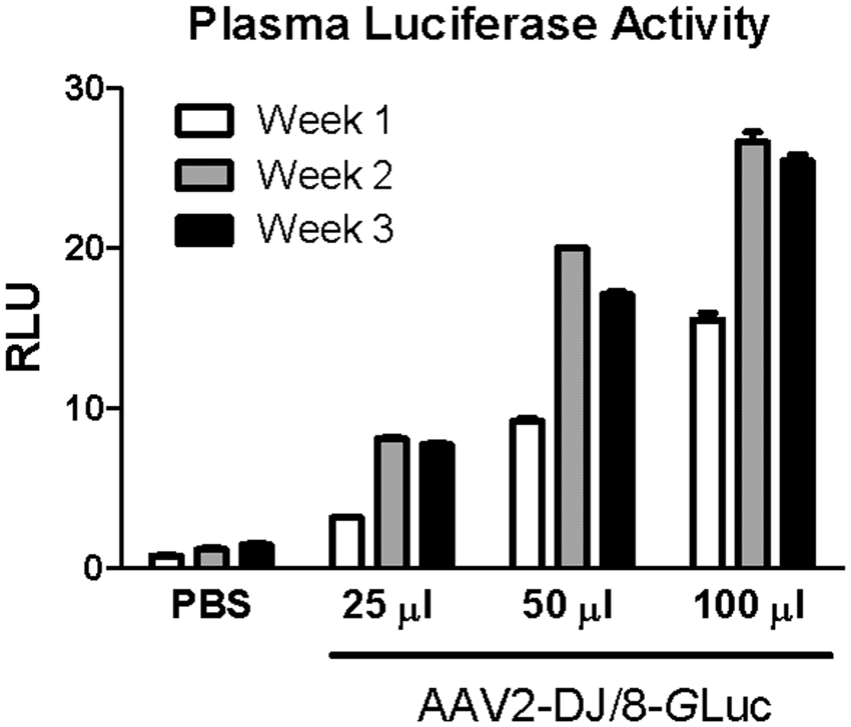
Relative plasma luciferase activity. Relative plasma luciferase activity of AAV2-DJ/8-*G*Luc (1.5 × 10^11^ vg/µl) tail vein injected male mice compared to PBS injected controls. Blood was collected from the tail vein at 1, 2, and 3 weeks after injection, and luciferase activity was measured. n = 4 per condition, SEM.

Surprisingly, our first *in vivo* experiment with retro-orbitally injected AAV2-DJ/8-shGlrx-mVenus following our purification protocol without discontinuous iodixanol gradient had little effect in mouse liver (data are not shown). We suspected impurities in the AAV preparations caused low-grade inflammation, which compromised virus infectivity and knockdown of Glrx in the liver, an effect also observed by others^58^. Thus, to quickly evaluate the onset and stability of gene expression, we generated an AAV expressing secreted *Gaussia* luciferase (AAV2-DJ/8-*G*Luc) and further purified the virus via a discontinuous iodixanol gradient, which resulted in a very pure AAV (>90%) as measured by silver staining. We injected the virus via the tail vein, and measured luciferase activity in tail vein bleeds. Groups of four male mice received either PBS or three different doses of AAV2-DJ/8-*G*Luc. Luciferase activity stabilized between week one and two and maintained comparable levels for at least three weeks. Also, luciferase activity increased dose-dependently (Fig. 5).

**Figure 6:**
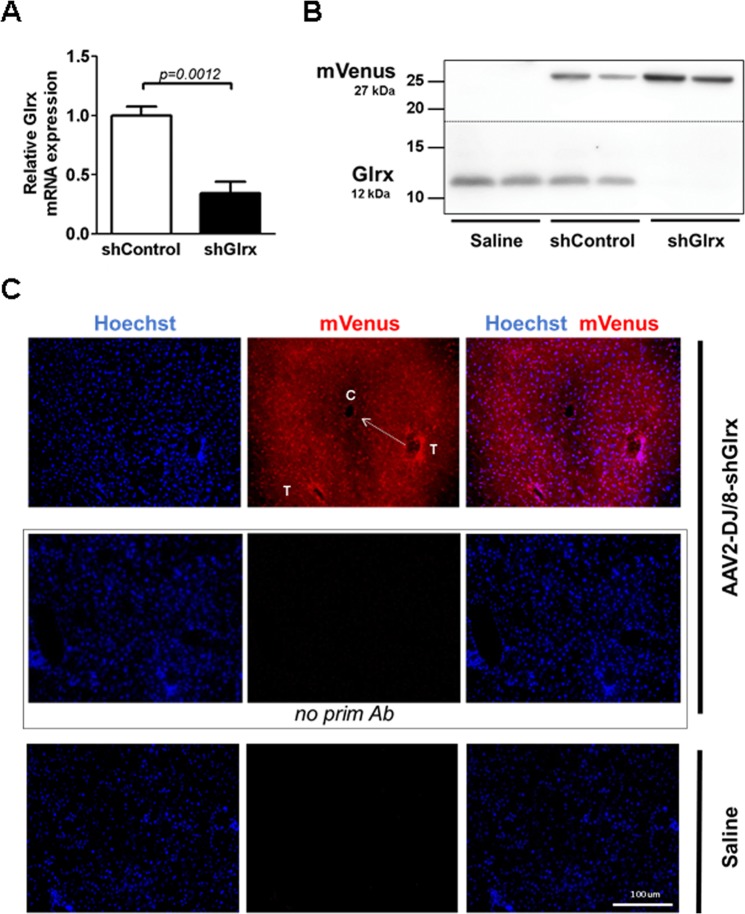
mRNA and protein expression of Glrx in mouse liver. (**A**) Glrx mRNA expression levels in livers of C57BL/6J mice two weeks after administration of AAV2-DJ/8-shControl-mVenus (shControl) or AAV2-DJ/8-shGlrx-mVenus (shGlrx). Gene expression was measured by RT-qPCR and the statistical analysis was performed using the two-tailed Mann-Whitney test. Statistical differences (n = 6–7; P < 0.05; SD) were compared to shControl. (**B**) Western blot analysis of the liver samples described in (**A**). The membrane (dotted line indicates the cut) was probed for mVenus and Glrx expression 2 weeks post AAV injection. (**C**) Liver sections from an AAV2-DJ/8-shGlrx-mVenus injected mouse showed a strong fluorescence signal of mVenus (top row, red color) in cytoplasm and nuclei. Nuclei were counterstained with Hoechst 33342. The mVenus signal was most intense around the triad (T) and faded towards (arrow) the central vein (**C**), showing zoning across the liver acinus. Neither liver sections of AAV2-DJ/8-shGlrx-mVenus injected mice, incubated with secondary antibody alone (middle row), nor saline-injected mice exhibited a significant mVenus signal at identical exposure time settings. Scale bar denotes 100 µm.

Subsequent purification of AAV2-DJ/8-shGlrx-mVenus via a discontinuous iodixanol gradient resulted in a 66% knockdown of Glrx mRNA in the liver compared to mice injected with AAV2-DJ/8-shControl-mVenus (Fig. 6A). Hepatic Glrx protein expression almost disappeared in AAV2-DJ/8-shGlrx-mVenus administered mice but remained unaffected in AAV2-DJ/8-shControl-mVenus injected mice (Fig. 6B).

We detected viral infection of the liver by immunohistology using a GFP-antibody that cross-reacts with mVenus, the yellow fluorescent protein variant co-expressed by AAV2-DJ/8-shGlrx-mVenus. Because retro-orbitally injected AAV2-DJ/8-shGlrx-mVenus entered the liver via the hepatic artery (Fig. 6C, top row), mVenus expression was highest around the triad (hepatic artery, bile duct, and portal vein; **T**). Viral particles decreased while traveling to the central vein, which explains the gradual decrease (**white arrow**) of mVenus expression towards the central vein (**C**), an effect referred to as zoning of the liver acinus. Liver sections of AAV2-DJ/8-shGlrx-mVenus injected mice stained with secondary antibody only (**middle row**), or saline-injected mice (**top row**) showed no mVenus expression.

### Testing AAVs in mouse skeletal muscle

**Figure 7:**
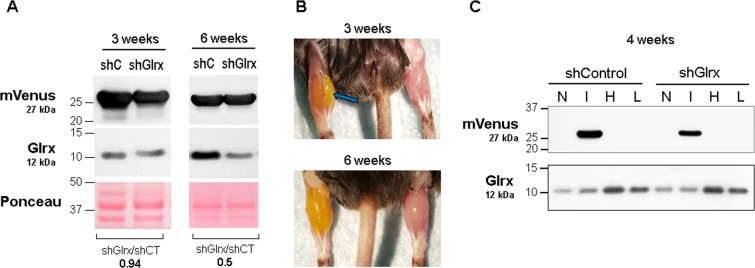
AAV2-6 mVenus transduced skeletal muscle. AAV2-6-shGlrx-mVenus *IM* injection induced in mVenus protein expression after 2 weeks but inhibition of Glrx expression occurred only after 6 weeks. (**A**) mVenus and Glrx expression in AAV-injected muscles. Mice were euthanized at 3 and 6 weeks after *IM* injection. shCT: AAV expressing short hairpin control. shGlrx: AAV expressing short hairpin RNA to Glrx. At 6 weeks, shGlrx inhibited Glrx expression to half of the control. Ponceau staining is shown as protein loading control. (**B**) *Upper panel*: The AAV-injected muscle turned yellow by mVenus expression and was swollen (**arrow**) compared to the non-injected muscle at 3 weeks. *Lower panel*: After 6 weeks, the muscle size appeared similar to the non-injected muscle. (**C**) mVenus and Glrx expression in other organs at 4 weeks after AAV *IM* injection. mVenus only expressed in the injected gastrocnemius muscle (**I**), but the AVV did not leak into the non-injected muscle (**N**), heart (**H**), or liver (**L**). AAV-shGlrx-injected muscles did not decrease Glrx protein expression at 4 weeks post-injection.

AAV injection into the skeletal muscle was more challenging than previously anticipated. Similar to the liver, the virus requires high purity and a high viral titer for injection of small volumes into the muscle. We examined the tissue-specific expression of AAV2-6-shGlrx-mVenus since this capsid has a better tissue tropism for skeletal muscle^59–62^. Also, using a different capsid further supports the general applicability of our protocol. After *IM* injection, we detected a high level of mVenus expression after 3 weeks in the injected gastrocnemius muscle (Fig. 7A, left) but not in the non-injected muscle, heart, or liver (Fig. 7C). These data demonstrate *IM* injection of AAV2-6 restricts the infection to the muscle.

Although AAV2-6 efficiently transduced mVenus expression in skeletal muscle and AAV2-DJ-shGlrx-mVenus suppressed Glrx expression in C2C12 cells (Fig. 4B,C), *in vivo* inhibition of muscle Glrx by AAV2-6-shGlrx-mVenus proved challenging. After 3–4 weeks of AAV2-6-shGlrx-mVenus *IM* injection, the protein level of Glrx in the muscle remained unchanged compared to control-AAV injected or non-injected muscle (Fig. 7A, left). The purity of AAVs was similar to that of AAV2-DJ/8-shGlrx-mVenus, which inhibited liver Glrx expression after 2 weeks. However, *IM* injection may cause local stress and inflammation of the muscle compared to intravenous injection. The muscle expressed mVenus highly and appeared yellowish and swollen after 3 weeks (Fig. 7B). After 6 weeks of AAV2-6-shGlrx-mVenus *IM* injection, the muscle inflammation resolved, and Glrx expression decreased in the AAV2-6-shGlrx-mVenus injected muscle compared to the AAV2-6-shControl-mVenus injected muscle (Fig. 7A,B).

## Discussion

We established an improved protocol that allows fast and efficient purification of AAVs independent of the serotype. Our protocol is composed of purifications from both HEK293T cell lysates and culture medium. Optimization of the culture medium demonstrated that excess glucose adversely affects viral production by promoting acidification, presumably through excessive glycolysis. As established for other cell types and viruses glutaminolysis is more critical to sustaining cell metabolism and robust virus production^31–35^. Glutamine feeds directly into the citric acid cycle to provide energy but also contributes to anaplerosis, a process to replenish the cycle with metabolites used in anabolic reactions such as amino acid synthesis. Therefore, we recommend the use of pH stabilized low glucose DMEM supplemented with stable glutamine (GlutaMAX).

Furthermore, the AAV samples with discontinuous iodixanol gradient purification improved the purity of AAVs. Using an AAV2-DJ/8-shGlrx-mVenus but omitting the iodixanol purification could not silence the expression of hepatic Glrx *in vivo*, suggesting contaminants cause inflammation and induce endogenous protein, whereas the same AAV after iodixanol purification efficiently attenuated the gene expression (data not shown). The results indicate the iodixanol purification as a necessary step to obtain AAVs that are suitable for *in vivo* use. Zolotukhin *et al*. also showed that purification with iodixanol was useful to obtain viruses of higher titer and purity compared to the cesium chloride ultracentrifugation method^23^. Because of minimal ionic and osmotic effects of iodixanol, gradient fractions after the final purification can be directly used without dialysis in cell and animal experiments. However, iodixanol in high amounts may lead to renal dysfunction. In our study, we obtained AAVs with a purity of greater than 90% in high yields of 10^10^–10^11^ vg/µl. Also, our method needs only seven days from transfection with the AAV plasmids until the final viral suspension for *in vivo* use. Using primers against the ITRs sequences for AAV titer determinations with qPCR may overestimate the viral particle content caused by the amplification of contaminating incomplete viral particles. The sequence coding for shRNAs may form stable DNA structures that mimic ITRs leading to early termination of viral single-stranded DNA (ssDNA) replication and generation of incomplete AAVs^63^. We applied a different shRNA sequence design, introduced by Miyagishi *et al*., to minimize the production of incomplete particles^19^. As our experiments in cultured cells and *in vivo* demonstrated, we obtained sufficient viral particles to suppress Glrx gene expression.

AAVs have been used to express transgenes *in vivo*, but *in vitro* applications are rare. However, as we show here using primary mouse hepatocytes and skeletal muscle-derived C2C12 cells, AAV2-DJ efficiently infects cells *in vitro*. The AAV2-DJ vector is a chimeric virus with a hybrid capsid from different wild-type AAVs, and predominantly shows high homology to wild-type AAV-2, AAV2-8, and AAV2-9, the three most efficient serotypes for mouse liver infection^10,64^. AAV2-DJ performs more effectively in various types of cells compared to the other eight primary AAV serotypes^10^ and is highly potent in mouse liver^64^. The variant AAV2-DJ/8 lacks a heparinbinding domain, which attenuates its efficiency *in vitro*, but broadens tissue distribution *in vivo*^10^ and can penetrate the central nervous system.

Different AAV serotypes control tissue tropism to a certain degree. Systemic administration of AAV2-DJ or AAV2-DJ/8 shows a favorable expression in liver^10,64^. Gene delivery applications to the muscle of dystrophic mice widely use the AAV2-6 vector, but the vascular delivery of AAV2-6 transduces both cardiac and skeletal muscles^59,60^. We demonstrated that *IM* injection of AAV2-6-shGlrx-mVenus resulted in mVenus expression in the muscle but not in the heart or liver, suggesting *IM-*injected AAV2-6 may transduce more selectively the skeletal muscle. The use of tissue-specific promoters in AAV-mediated gene expression system, such as albumin (Alb) and thyroxine-binding globulin (TBG), can improve targeted AAV-mediated gene expression^5^. Exchanging promoters to target a specific tissue or cell type is desirable for AAV-mediated gene therapy.

Since systemically administered AAVs may affect other organs, we performed *IM* injection to restrict the AAV-mediated gene suppression of Glrx to the skeletal muscle. AAVs are widely used for gene delivery to the muscle^58,65^, and intramuscular injection can be successfully delivered and stably expressed over five months in the mouse muscle^66^. We detected mVenus expression in the muscle already after 3 weeks. However, suppression of muscle Glrx expression by AAV2-6-shGlrx took longer, and we detected it decreased after 6 weeks. This long delay was unexpected because the systemically injected AAV2-DJ/8-shGlrx-mVenus markedly suppressed liver Glrx expression after 2 weeks. We speculate that Glrx protein turnover in the muscle may be slower than in the liver.

Furthermore, the injected muscle looked yellowish and swollen, and the AAV may induce inflammatory genes in the muscle at 3 weeks. The injected AAV2-6-shGlrx-mVenus also expresses mVenus as a marker. It is important to remark that fluorescent proteins such as GFP, and variants thereof, may produce superoxide and cause oxidative stress^67^. Notably, for therapeutic purposes of minimizing inflammation and off-target effects, we believe that mVenus should be omitted. Glrx is known as a NF-kB responsive gene^68^. We speculate that AAV-induced inflammatory responses in the muscle may activate the NF-kB pathway and upregulate Glrx expression^68^, counteracting the inhibition by shGlrx. Clerk *et al*. also observed inflammatory cell infiltration into the injected muscle up to one month after *IM* AAV injection^66^. Even though AAVs are supposed to cause less inflammation compared to adenovirus, one should take into account inflammatory responses caused by AAV injection. Also, intravascular injection of AAVs causes an immune response to the AAV capsid and inflammation in muscles, which is improved by a short course of an immunosuppressant^60^. Clinically, naturally-occurring neutralizing antibodies to AAV capsids can attenuate AAV-mediated gene therapy. Interestingly, in the presence of a neutralizing antibody to AAV2-8 capsid, *IM* injection of AAV2-8 still delivers the transgene to muscles, but abolishes gene expression in liver^69^, suggesting the usefulness of *IM* injection to induce transgene expression in muscles of people with naturally-occurring antibodies to AAVs.

In summary, we presented a refined, rapid, and economical protocol to produce and purify AAVs, which efficiently transduce genes *in vitro* and *in vivo*. Iodixanol purification is helpful to eliminate contaminants and thus improve AAV purity, diminish inflammation, and improve viral transduction *in vivo*.

## Supplementary information

AAV Calculations Spreadsheet

Protocols, Original Blots, and Drawings

Vector Maps and Sequences
