# Supplementary material for: Production of adeno-associated virus vectors for in vitro and in vivo applications
Source: Sci Rep. 2019 Sep 19;9:13601. doi: 10.1038/s41598-019-49624-w (PMC6753157; doi:10.1038/s41598-019-49624-w)

Supplemental Information

Production of adeno-associated virus vectors for *in vitro* and *in vivo* applications

Toyokazu Kimura[+*$], Beatriz Ferran[+*], Yuko Tsukahara*, Qifan Shang*, Suveer Desai*, Alessandra Fedoce*, David Richard Pimentel[#], Ivan Luptak[#], Takeshi Adachi[$], Yasuo Ido[$], Reiko Matsui[+*], and Markus Michael Bachschmid[+*]

*Vascular Biology Section and [#]Cardiology, Whitaker Cardiovascular Institute, Boston University School of Medicine
[$]Cardiovascular Medicine, National Defense Medical College, Japan

\+ Authors have equally contributed

Supporting Protocols

Plasmid DNA Maxi Preparation

Reagents

NEB stable competent cells
SOC medium
Resuspension buffer: 50 mM Tris·Cl, pH 8.0, 10 mM EDTA, 100 µg/ml RNase A (AppliChem). Store buffer in a plastic container at 4 ºC
Note: *Use thymolphthalein as a pH indicator for visual control of cell resuspension and lysis. Prepare a 1000x stock (40 mg/mL in ethanol) and add to the resuspension buffer.*
Lysis buffer: 200 mM NaOH, 1% SDS (w/v). Store buffer in plastic container
Binding buffer: 3.0 M potassium acetate, pH 5.5
Equilibration buffer: 750 mM NaCl, 50 mM MOPS, pH 7.0, 15% isopropanol (v/v), 0.15% Triton® X-100 (v/v)
Wash buffer: 1.0 M NaCl, 50 mM MOPS, pH 7.0, 15% isopropanol or ethanol (v/v)
Elution buffer: 1.25 M NaCl, 50 mM Tris·Cl, pH 8.5, 15% isopropanol (v/v)
Regeneration buffer 1: 1N HCl, 0.15% Triton® X-100 (v/v)
Regeneration buffer 2: 3N NaCl
TE buffer: 10 mM Tris·Cl, pH 8.0, 1 mM EDTA
70% ethanol (v/v) (Molecular Biology grade)
Isopropanol (Molecular Biology grade)
Maxi-Prep anion exchange columns (Epoch Life Science)
Zymo-Spin III-P with 15 ml and 50 ml reservoir (ZymoResearch)
LB medium: 10 g/l tryptone, 5 g/l yeast extract, 5 g/l NaCl, pH 7.0.
LB plates: 1.5 g/l agar in LB medium
Selective LB medium or LB plates: LB medium or LB plates supplemented with the appropriate antibiotic
Filter paper and funnel or filter holder: e.g., 2.5 cm SWINNEX® syringe filter holder (Millipore) with P8 filter paper (Thermo Fisher)

Tips

- Use a recombinase deficient *E. coli* strain such as NEB stable to avoid recombination or damage to viral plasmids containing ITRs.
- Transduce NEB stable cells according to the manufacturer's instructions. Use the high-efficiency transformation protocol for cloning and the 5-minute protocol for purified plasmids.
- Grow *E. coli* in LB selection plates and LB selection medium between room temperature and 30 ºC. Higher temperatures decrease plasmid yield and increase the probability for plasmid recombination.
- Use LB medium only. Richer media promote recombination and reduce plasmid copy numbers.

- Store plasmids in TE buffer at 4 ºC.
- Do not vortex plasmid solutions to avoid DNA shearing.
- Always streak out bacteria and check DNA integrity by restriction enzyme analysis.
- Prewarmed buffers, as indicated in the protocol, solubilize DNA better and increase the plasmid yields.

Procedure

1. Pick a single colony from a freshly streaked LB selective plate and inoculate a starter culture of 10 ml of selective LB medium.
2. Incubate at room temperature with shaking (150-250 rpm) overnight or until a sufficient cell density is reached.
3. Add 0.5-2 ml of starter culture to 250 ml of selective LB medium, content in a baffled shake flask.
4. Grow at room temperature overnight with shaking until the OD600 of the bacterial culture reaches a value higher than 2.0.
5. Split the culture evenly into two centrifuge tubes and harvest *E. coli* by centrifugation at 3,500 x g in a fixed angle rotor for 10 min at ~4 °C.
6. Suspend both bacterial pellets each in 10 ml of resuspension buffer.
7. Add 10 ml of lysis buffer, mix thoroughly by inverting 4–6 times, and incubate at room temperature for 4 min on a rocker under gentle rocking.
8. Add 11 ml of chilled binding buffer, mix immediately and thoroughly by vigorously inverting 4–6 times, and incubate on a rocker for 10 min under gentle rocking.
9. Centrifuge at 2,500 x g for 15 min at 4 °C.
10. Remove the top white fluffy layer and promptly add the plasmid DNA-containing supernatant into a filter paper.
11. Collect the filtered bacterial lysate into clean tubes.
12. Equilibrate the maxi column by applying 10 ml of equilibration buffer, and allow the column to empty by gravity flow.

    Note: *Skip step 12, when using regenerated columns.*

13. Apply ~30 ml (one tube) of the lysate from step 8 to the column and allow it to enter the resin by gravity flow.
14. Wash the column with 30 ml of wash buffer.

    Note: *This step removes RNAs to free column capacity for plasmid binding and increase plasmid yield.*

15. Repeat step 10 and 11 with the second tube.
16. Wash the column with 2 x 30 ml of wash buffer.
17. Elute the DNA with 15 ml of elution buffer, preheated to 50-60 °C.
18. Precipitate the DNA by adding 12 ml of room temperature isopropanol. Mix immediately and filter the solution through a Zymo-Spin III-P filter (vacuum or centrifuge).

    Alternatively: *Zymo columns are fast to collect, wash, and elute DNA. Alternatively, perform an isopropanol DNA precipitation and collect plasmid DNA by centrifugation.*

16. Wash the Zymo filter with 2 x 800 µl of 70% ethanol.
17. Spin filter dry at 10,000 x g for 1 min and place it into a new sterile microcentrifuge tube.
18. Add 300 µl of TE buffer prewarmed at 50-60 °C to the filter, incubate for 5 min, and spin at 10,000 x g for 1 min.
19. Measure the OD with Nanodrop and run a restriction enzyme digestion to check the plasmid integrity.

Zymo-Spin III-P filters regeneration

1. Wash the Zymo filter with 3 x 800 µl of TE buffer.
2. Wash the Zymo filter with 3 x 800 µl of 70% ethanol.

Anion exchange column regeneration

1. Store the column overnight in 30 ml of regeneration buffer 1.

   Note: *HCl destroys residual DNA.*

2. Wash the column with 30 ml of regeneration buffer 1.
3. Wash the column with 2 x 30 ml of regeneration buffer 2.
4. Wash the column with 30 ml of ddH_2O.
5. Wash the column with 2 x 30 ml of equilibration buffer.

AAV production protocol

Reagents

Growth medium: DMEM (Thermo Fisher, 4.5 g/l glucose, L-glutamine, pyruvate), 5% FBS (Atlanta Biologicals), 1x Glutamax (Thermo Fisher), 1x penicillin/streptomycin (Thermo Fisher).
Production medium: DMEM (Thermo Fisher, 1 g/l glucose, L-glutamine, pyruvate); 1% FBS (Atlanta Biologicals), 1x Glutamax (Thermo Fisher), 1x penicillin/streptomycin (Thermo Fisher), 10 mM HEPES (Thermo Fisher), Add 0.075% sodium bicarbonate (Thermo Fisher).
PBS for cell culture (Thermo Fisher)
PEI solution: 5 µg/µl polyethylenimine, linear, MW~25,000 (Polysciences) in 0.1 N HCl. Can be aliquoted and stored at -80 ºC.
5x PEG/NaCl solution: 40% PEG 8000 (w/v), 2.5 M NaCl
TNE buffer: 100 mM Tris·Cl, pH 8.0, 150 mM NaCl, 20 mM EDTA
0.5 M EDTA; pH 8.0 (Thermo Fisher)
AAV releasing solution (110 mM citrate buffer pH 4.2): 55 mM citric acid, 55 mM sodium citrate, 800 mM NaCl
AAV neutralizing solution: 2 M HEPES; pH 8.0
Chloroform (Molecular Biology grade, Thermo Fisher)
20% $(NH_4)_2SO_4$ solution (w/w)
50% PEG 8000 (w/w)
10% Pluronic F68 (Sigma)
OptiPrep (60% (w/v) Iodixanol (Axis-Shield PoC AS)
10x PBS-MK: 10 mM $MgCl_2$, 25 mM KCl, in 10x PBS
Salt solution: 2 M NaCl in PBS-MK
Solution 54% (w/v): 9 vol. of OptiPrep, 1 vol. 10x PBS-MK
Solution 40% (w/v): 4.0 vol. solution 54%, 1.4 vol. PBS-MK
Solution 25% (w/v): 2.5 vol. solution 54%, 2.9 vol. PBS-MK
Solution 15% (w/v): 1.5 vol. solution 54%, 1.2 vol. PBS-MK, 2.7 vol. salt solution
0.01 µg/ml Phenol red solution
Beckman Coulter Quick-Seal™ centrifuge tubes
Type 70.1 Ti Beckman or equivalent rotor
18 G short, 18 G long (spinal) and 25 G needles and syringes

Procedure

Culture HEK293T cells in growth medium until reaching 50-70% confluence. Passage or transfect cells.

Important: *It is recommended not to let HEK293T become too confluent because cells may differentiate, decrease transfection efficiency, and viral production.*

Day 0: Transfection

Prepare the transfection mix with DNA and the PEI in a ratio 1:3 (w/w).

> ***Note:*** *According to the size of the culture dish, use the AAV excel spreadsheet to adjust the medium volumes and calculate DNA and PEI amounts.*

1. Before transfection (2-4 hours), replace the culture medium with fresh growth medium.
2. Dilute all plasmid DNA in PBS to prepare solution A and mix well.
3. Dilute the PEI solution in PBS to prepare solution B and mix well.
4. Combine solutions A and B and gently vortex to mix.
5. Allow the mixture to stand at room temperature for at least 20 min to let polycomplexes form.
6. Add the transfection mix to the cells in a dropwise manner.
7. Carefully mix and incubate at 37 °C overnight.

Day 1: Change to the production medium

Replace the transfection medium with the proper volume of production medium (see AAV excel spreadsheet) and incubate at 37 °C.

Day 3: First collection of the AAV-containing medium

1. Carefully collect the AAV-containing medium in appropriate centrifuge tubes.
2. Add fresh production medium to the cells and incubate at 37 °C.
3. Centrifuge the collected medium at 2,000 x g for 10 min at 4 °C to remove the cell debris, and transfer the supernatant into a new tube.
4. Measure the volume of the AAV-containing supernatant and add ¼ volumes of 5x PEG/NaCl solution (proportion 4:1, supernatant: PEG/NaCl).
5. Mix well by inverting and incubate overnight at 4 °C.

> ***Note:*** *AAVs are stable up to 2 days in this solution. Use a pipette to measure the PEG/NaCl volume accurately.*

Day 4: PEG/NaCl precipitation

1. Centrifuge the AAV-PEG/NaCl mixture at 2,500 x g for 1 h at 4 °C and carefully remove the supernatant by aspiration.

   > ***Note:*** *Be sure to remove all the supernatant. Spin down and aspirate again if necessary.*

2. Resuspend by vortexing the beige to white viral pellet in the proper volume of cold TNE buffer.

   > ***Note:*** *Do not resuspend the pellet by pipetting because this may shear the viral particles and decrease the final AAV yield. Let the pellet sit at 4 °C and mix every so often until it is entirely suspended.*

3. Store at -80 °C the viral suspension for future use (aliquot), or combined it with the second viral suspension of day 6.

> **Note:** *In some cases, PEG may increase viral transduction in cell culture experiments. However, if desired, remove PEG by chloroform extraction.*

Day 5: Final collection of the AAV-containing medium and cells

1. To detach HEK293T cells, add 0.5 M EDTA (pH 8.0) in a 1:80 ratio of the production medium.
2. Allow the plates to stand at room temperature for 10 min.
3. Carefully collect the mixture of medium and detached cells and centrifuge at 750 x g at 4 °C for 15 min.
4. Transfer the AAV-containing supernatant into a clean tube and keep at room temperature.
5. Loosen the cell pellet by tapping and add 1 ml of PBS to wash the cells.

> **Note:** *If the cell pellet is not sufficiently suspended, the efficiency of extraction may decrease.*

6. Mix by vortexing and centrifuge at 1,000 x g at 4 °C for 15 min.
7. Thoroughly collect the supernatant, combine with the AAV-containing supernatant from step 3, and proceed as described in 'First collection of the AAV-containing medium' (steps 4 and 5).

> **Note:** *Confirm complete removal of PBS before proceeding; particle isolation may be affected by residual supernatant due to acidity of the citrate buffer.*

5. Loosen the cell pellet by tapping and add the proper volume of AAV releasing solution (see AAV excel spreadsheet).
6. Resuspend the cell pellet by vortexing for 15 seconds and allow standing at room temperature for 10 min.
7. Vortex for 15 s and centrifuge at 14,000 x g at 4 °C for 15 min.
8. Collect the supernatant in a new tube.

> **Note:** *The mixture can be stored at -80 °C. Thaw quickly in a 37 °C water bath before continue. Phenol red can be added to facilitate further titration.*

9. Measure the volume of the supernatant and, under continuous mixing, add ~⅕ volumes of AAV neutralizing solution dropwise. The final pH should be around 8.0 and proceed as described in 'First collection of the AAV-containing medium' (steps 4 and 5).

> **Note:** *If phenol red was added, the solution should change from yellow (acid pH) to pink (pH ~ 8.0) color.*

Day 6: Perform PEG/NaCl precipitation, chloroform extraction, aqueous two-phase partitioning, and iodixanol discontinuous gradient centrifugation.

PEG/NaCl precipitation

1. With the AAV-PEG/NaCl mixtures from medium and cell lysate, proceed as described for 'Day 4: PEG/NaCl precipitation' (step 1 and 2).
2. Combine the viral suspension from medium and cell lysate with the viral suspension obtained at day 4.

Chloroform extraction

1. Measure the volume of the total viral suspension and add an equal volume of chloroform (proportion 1:1 (v/v)).
2. Vigorously vortex for 2 min and centrifuge at 1,000 x g for 5 min at room temperature.
3. Collect the top layer (AAV-containing supernatant) in a new tube and discard the bottom layer (chloroform).

   Caution: *Treat chloroform containing solutions as hazardous waste!*

4. Place AAV-containing supernatant under a chemical hood and let remaining chloroform evaporate for 30 min.

   Note: *After complete evaporation of the chloroform, the AAV-containing supernatant can be used for in vitro studies. Aliquot the AAVs and store at -80 °C.*

Aqueous two-phase partitioning

1. Weigh the AAV-containing supernatant.
2. Per 1 g of the AAV-containing supernatant, add 5 g of 20% $(NH_4)_2SO_4$ solution and 1.5 g of 50% PEG 8000 solution.

   Important: *Use weight/weight ratios.*

3. Vigorously vortex the mixture for 2 min and let sit for 20-30 min at room temperature.
4. Centrifuge the two-phase mixture at 2,500 x g for 15 min at room temperature for prompt phase separation.
5. Remove the top layer and the interphase by aspiration and transfer the clear bottom phase (AAV-containing $(NH_4)_2SO_4$ solution) into a new tube.

   Note: *Some proteins salt out and form a precipitate at the bottom. The unprecipitated virus resides in the bottom soluble phase. All other proteins partition into inter- and top PEG phase.*

6. (Optional) If desired, exchange buffer with an Amicon™ 100 kDa cutoff centrifuge filter and store concentrated virus in the presence of 0.01% Pluronic F68.

   Note: *Before use, rinse the Amicon centrifuge filters with 0.01% Pluronic F68 to avoid AAV adsorption to plastic surfaces.*

Iodixanol discontinuous gradient centrifugation

Note: *This step is highly recommended for in vivo studies as it removes empty capsids and contaminant proteins from the final AAV suspension.*

1. If the volume of the AAV-containing $(NH_4)_2SO_4$ solution is higher than 5 ml, reduce the volume with an Amicon™ 100 kDa cutoff centrifuge filter.

> ***Note:*** *Before use, rinse the Amicon centrifuge filters with 0.01% Pluronic F68 to avoid AAV adsorption to plastic surfaces.*

2. Form an Iodixanol step gradient. Using a long blunt metal cannula (0.8 mm i.d.) attached to a syringe, add the AAV-containing solution to the ultracentrifuge tube.
3. Underlying the viral fluid, add 3 ml of 15% iodixanol solution directly in the bottom of the tube. In the same way, add 2 ml of 25% iodixanol solution, followed by 1 ml of 40% iodixanol and 1 ml of 54% iodixanol gradient solutions.

> **Note:** *Stain the gradient solutions alternately with phenol red (0.01 µg/ml) to distinguish the gradient layers. Centrifugation accelerates iodixanol diffusion, dissolving the clear boundaries of the iodixanol step gradient (**Fig. 2**). Top loading is discouraged and results in diffuse bands.*

4. Remove air bubbles by carefully tapping the ultracentrifuge tube and seal properly.
5. Ultracentrifuge at 350,000 x *g* for 2 h at 25 °C.

> ***Important:*** *Use slow acceleration and deceleration (up to and below 2,000 rpm) or turn the brake off below 2,000 rpm. Use k-factor conversion (see AAV spreadsheet) if rotor or centrifuge cannot reach the required g-force.*

> ***Note:*** *This protocol has been optimized for the Type 70.1 TI Beckman-Coulter rotor and centrifuge tubes. Use other fixed angle rotors only after k-factor conversion to ensure proper separation of AAVs from debris (see spreadsheet or Beckman Coulter publications) and adjust the volume of the lysate and gradient solutions proportionally.*

> ***Note:*** *Lower temperatures will increase the iodixanol viscosity and affect AAV separation!*

6. Puncture the bottom and the top of the ultracentrifuge tube with two 18 G needles.
7. Collect dropwise the whole gradient from higher to lower density, in 0.4-1 ml fractions.

> ***Note:*** *Collect the 40% iodixanol layer (AAV-enriched layer) in three fractions of 0.4 ml. Collect the remaining layers in 1 ml fractions. Alternatively, aspirate the 40% layer right below the 40%-54% interface with a syringe.*

8. Measure the AAV titer and the purity of all fractions by qPCR and SDS-PAGE combined with silver staining.
9. If desired, exchange buffer with an Amicon™ 100 kDa cutoff centrifuge filter to remove the iodixanol from the AAV-enriched fractions, and store concentrated virus in the presence of 0.01% Pluronic F68.
10. Store AAV suspensions aliquoted at 4 °C for a short time or at -80 °C for more extended periods.

Fast Silver Staining

Reagents

10% Acrylamide/Bis Acrylamide SDS-PAGE gel
Tris-glycine-SDS running buffer
Molecular weight marker (Precision Plus^{TM} Protein Standards-All Blue)

BSA standards: serial dilutions ranging from 3.1 to 12.5 ng/µl
Reducing sample buffer: 2.5% β-mercaptoethanol (v/v) in NuPAGE LDS sample buffer (4x) (Invitrogen)
Fixation solution: 30% ethanol (v/v), 10% glacial acetic acid (v/v)
Enhancing solution: 0.02% $Na_2SO_2O_3 \cdot 5H_2O$ (w/v)
Silver solution: 0.2% $AgNO_3$ (w/v) (from 3% $AgNO_3$ stock solution, Polysciences)
Developing solution: 0.05% formaldehyde (v/v), 0.000016% $Na_2S_2O_3 \cdot 5H_2O$ (w/v), 3% Na_2CO_3 (w/v)
Stop solution: 114 mM citric acid

Procedure

1. Prepare samples and BSA standards in reducing sample buffer, use a dilution series of BSA (31 to 125 ng/per lane).
2. Load the samples in a 10% Acrylamide/Bis-Acrylamide SDS-PAGE gel and perform the protein electrophoresis in Tris-glycine-SDS running buffer until the desired separation of the molecular weight marker bands.
3. Incubate the gel in fixation solution for at least 30 min. The gel can be left overnight in this solution and further processed the next day.
4. Rinse the gel twice in 20% (v/v) ethanol, 10 min for each wash, and then twice in water, 10 min for each wash.
5. Sensitize the gel in enhancing solution for 1 min.
6. Rinse the gel twice in water, 1 min for each wash.
7. Incubate the gel in silver solution for 20 min.

   Note: *Incubation can last from 20 min to 2 h without any real change in the quality of the result.*

7. Discard the silver solution, rinse the gel with distilled water and again with a small volume of developing solution.
   Caution: *Treat the silver nitrate solution as hazardous waste.*
8. Soak the gel in fresh developing solution until bands show desired intensities (~1-3 min).

   Note: *Development may continue for some time after adding the stop solution.*

9. Incubate the gel in stop solution for at least 30 min up to 2 h.
10. Wash the gel 10 min with distilled water.

    Note: *Gels can be stored in distilled water for several days.*

11. Silver staining results in three specific bands of VP1 (90 kDa), VP2 (72 kDa), and VP3 (63 kDa) that are distinct from bovine serum albumin (66 kDa). Quantify VP3 to estimate the viral titer using, e.g., the ImageJ software and $c=4.977x10^{-9}$ ng of VP3 per AAV particle (mean value of AAV2 $4.987x10^{-9}$ ng and AAV9 $4.967x10^{-9}$ ng as previously reported) [Johnson, Becerra, Kohlbrenner].
12. Calculate the protein amount of VP3 from linear regression analysis of the BSA standards. Dividing the protein amount of VP3 by the constant c yields the number of AAV particles loaded per gel lane.

1. *Johnson, F. B., Ozer, H. L. & Hoggan, M. D. Structural proteins of adenovirus-associated virus type 3. J. Virol. 8, 860–63 (1971).*
2. *Becerra, S. P., Koczot, F., Fabisch, P. & Rose, J. A. Synthesis of adeno-associated virus structural proteins requires both alternative mRNA splicing and alternative initiations from a single transcript. J. Virol. 62, 2745–54 (1988).*
3. *Kohlbrenner, E. et al. Quantification of AAV particle titers by infrared fluorescence scanning of Coomassie-stained sodium dodecyl sulfate-polyacrylamide gels. Hum. Gene Ther. Methods 23, 198–203 (2012)*

AAV titration by qPCR

Reagents

DNase I: 2000 units/ml DNase I (AppliChem), in 10 mM Tris·Cl, pH 7.6, 2 mM $CaCl_2$, 50% g lycerol
10x DNase buffer: 100 mM Tris·Cl, pH 7.6, 25 mM $MgCl_2$, 5 mM $CaCl_2$
2x Capsid lysis buffer: 0.5 M NaOH, 0.2 M EDTA
ITR-Fwd: Primer for forward ITR sequence (5'-GGAACCCCTAGTGATGGAGTT-3')
ITR-Rev: Primer for reverse ITR sequence (5'-CGGCCTCAGTGAGCGA-3')

> ***Note:*** *Although, using a primer pair to amplify the ITR sequences could be a universal way to titer different AAV genomes, it may overestimate the real titer of the preparations. To use specific primers against the region of interest (e.g. short hairpin cassette) is encouraged.*

PowerUp™ SYBR™ Green Master Mix (Applied Biosystems, Foster City, CA)

Procedure

1. Remove extra-viral DNA by digestion with DNase I.
   a. Mix 2 µl of viral suspension, 2 µl of 10x DNase I buffer, 1 µl of DNase I, and 15 µl of nuclease-free water.
   b. Incubate at 37 ºC for 15 min.
2. Release the viral DNA by alkaline lysis.
   a. Add 20 µl of the 2x Capsid lysis buffer to the previous reaction and mix well.
   b. Incubate at 95 ºC for 10 min.
   c. Stop the lysis reaction by adding 10 µl of 0.5 M Tris-HCl (pH 8.0).
   d. Prepare a 50-fold dilution of the lysis reaction using nuclease-free water.
3. Quantify the viral DNA by qPCR.
   a. Using a plasmid containing ITR sequences, prepare 5-fold serial dilutions ranging from $1x10^9$ to $6.4x10^4$ dsDNA molecules/µl.

Note: To determine the dsDNA molecule number per ng, open the sequence of the plasmid using the SnapGene Viewer software (free for Windows and Mac platforms). Select from the "Tools" pulldown menu "Show DNA Calculations." The pop-up window will provide the MW and the conversion of ng into plasmid copies.

As the viral genome consists of ssDNA, the detection of one molecule of plasmid DNA (dsDNA) equals two AAV vector genome (vg) copies.

b. In a final volume of 10 µl, mix 1 µl of the template (standard dilution or 50-fold sample dilution), 100 nM of ITR-Fwd, 340 nM of ITR-Rev, and 5 µl of the PowerUp™ SYBR™ Green Master Mix.

   Note: *Prepare each reaction at least by duplicate.*

c. Perform the PCR reaction under the following conditions: UDG treatment at 50 ºC for 2 min and polymerase activation at 95 ºC for 2 min, followed by 40 cycles of denaturation at 95 ºC for 3 s, and annealing/extension at 60 ºC for 30 s.

d. To obtain the final titer of the sample (expressed in vg/µl), multiply by 2500 the value of viral genome copies quantified by interpolation in the standard curve.

Supplemental Information

Original Western Blots and Drawings

Production of adeno-associated virus vectors for *in vitro* and *in vivo* applications

Toyokazu Kimura[+*$], Beatriz Ferran[+*], Yuko Tsukahara*, Qifan Shang*, Suveer Desai*, Alessandra Fedoce*, David Richard Pimentel[#], Ivan Luptak[#], Takeshi Adachi[$], Yasuo Ido[$], Reiko Matsui[+*], and Markus Michael Bachschmid[+*]

*Vascular Biology Section and [#]Cardiology, Whitaker Cardiovascular Institute, Boston University School of Medicine
[$]Cardiovascular Medicine, National Defense Medical College, Japan

\+ Authors have equally contributed

Figure 3_panel A

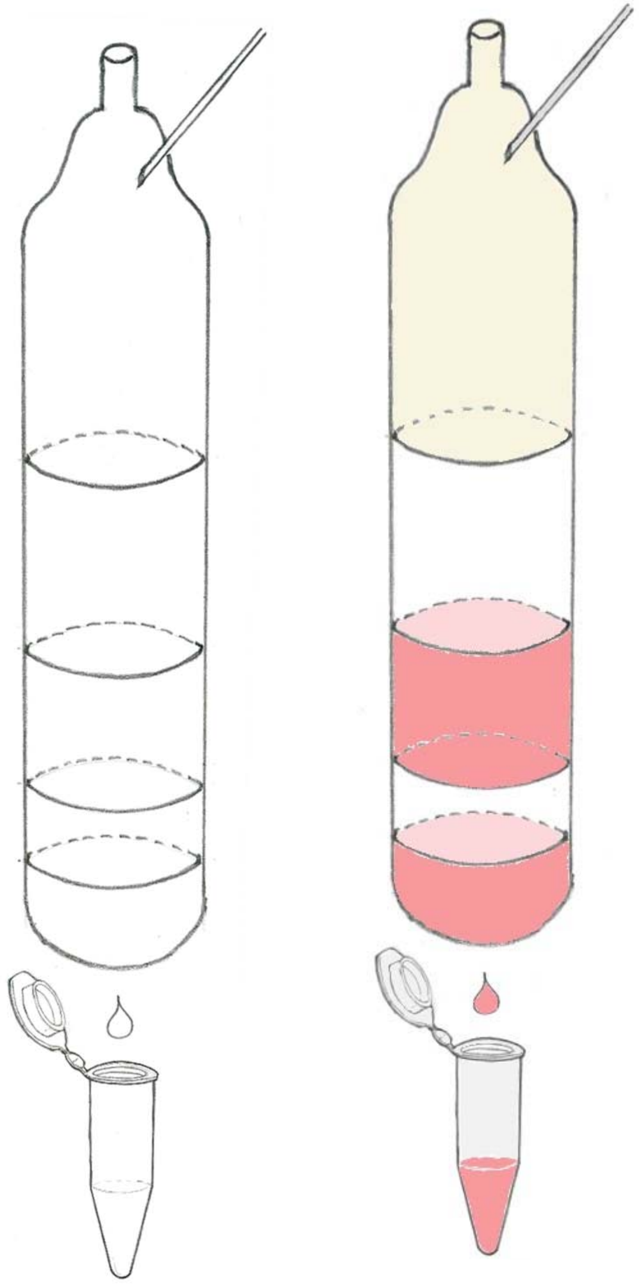

Figure 3_panel C

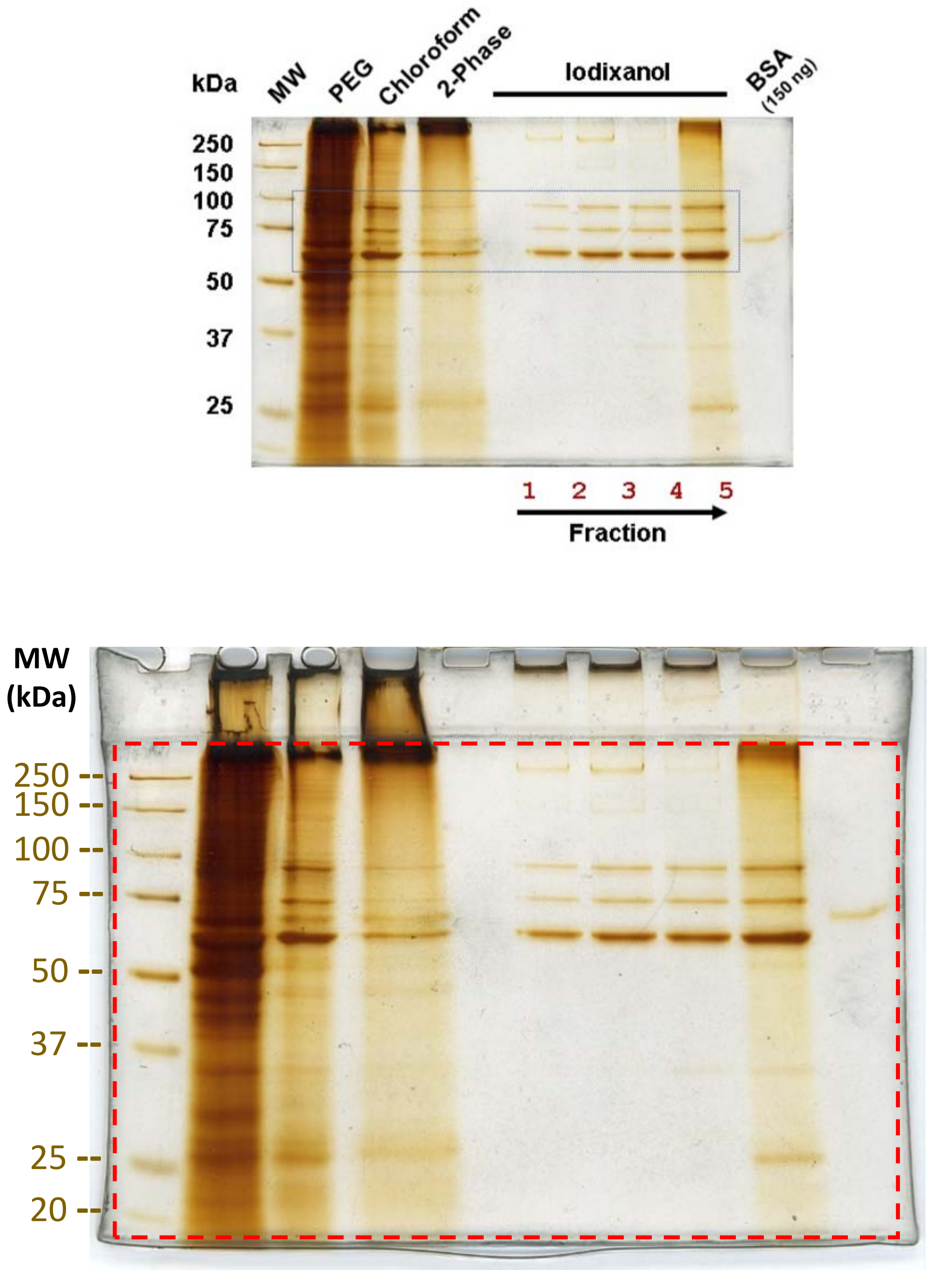

Figure 4_panel C

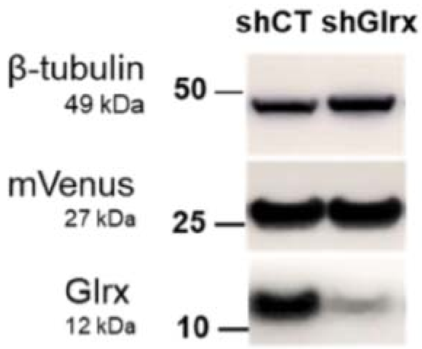

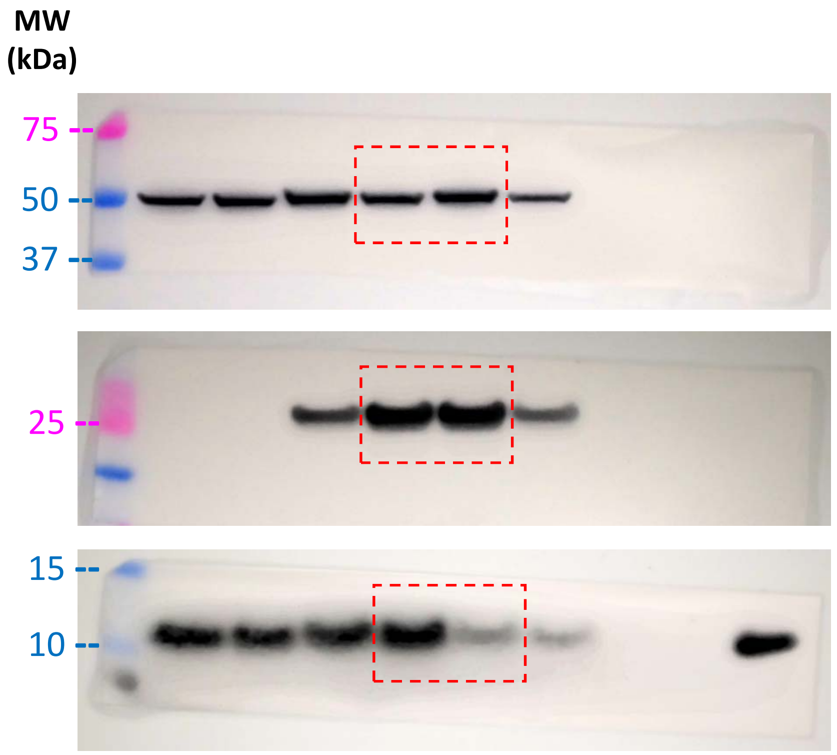

Figure 6_panel B

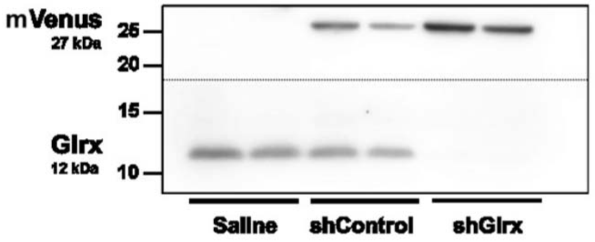

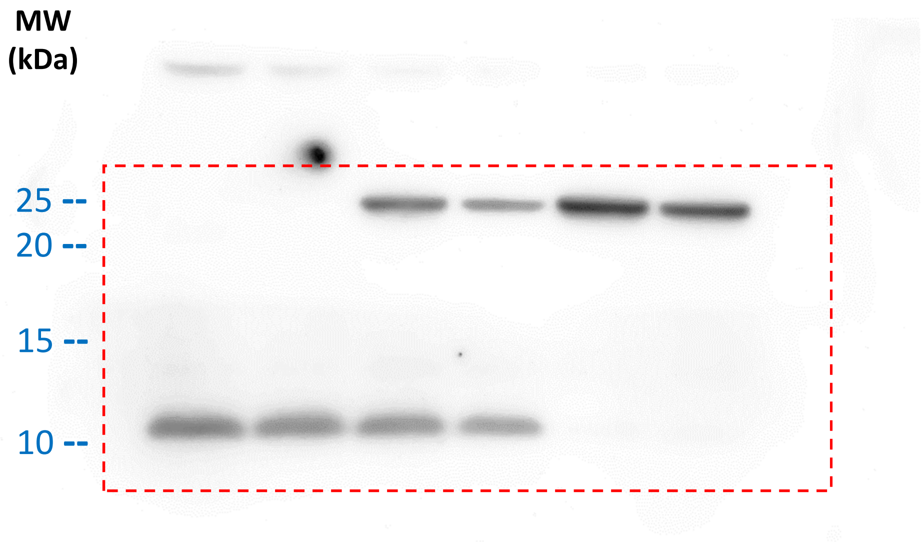

Figure 7_panel A

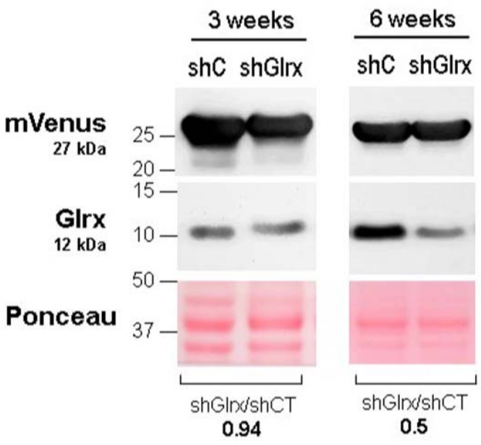

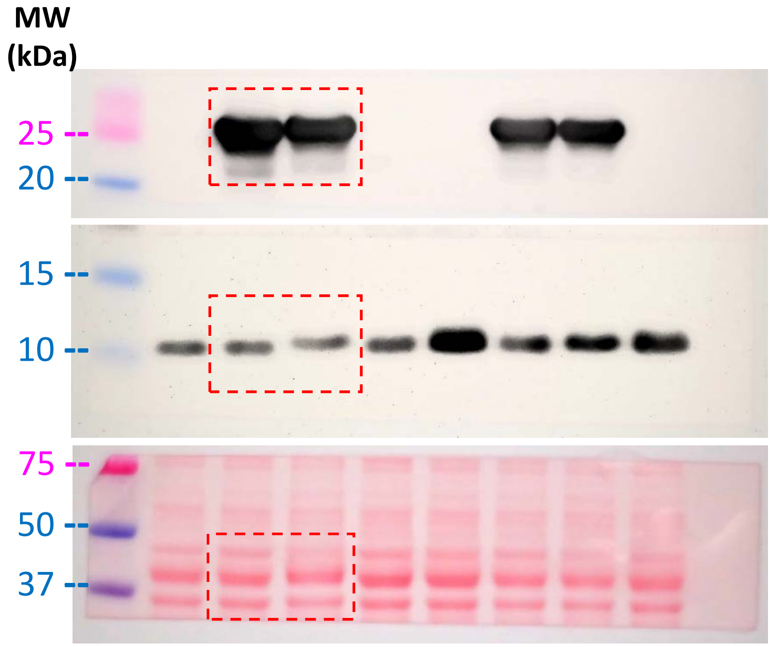

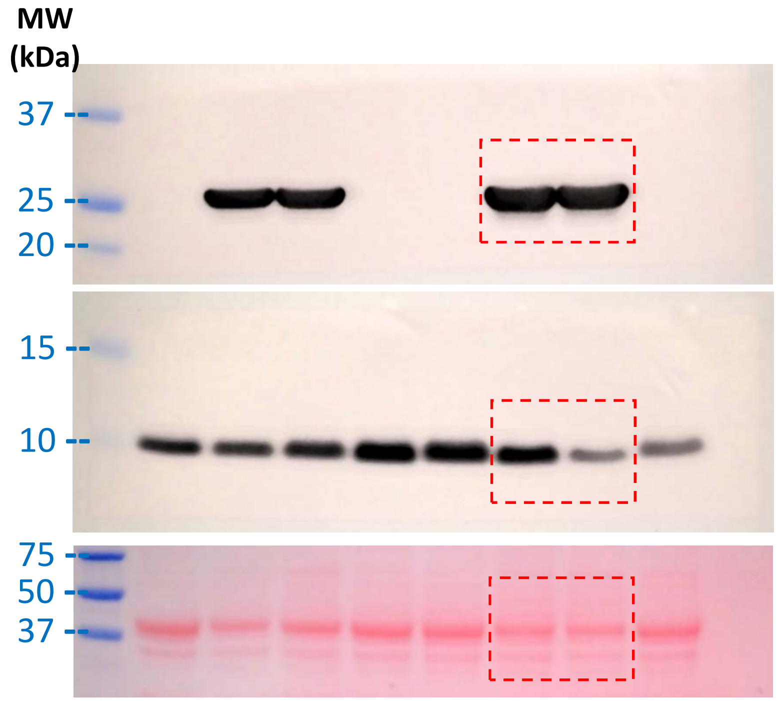

Figure 7_panel C

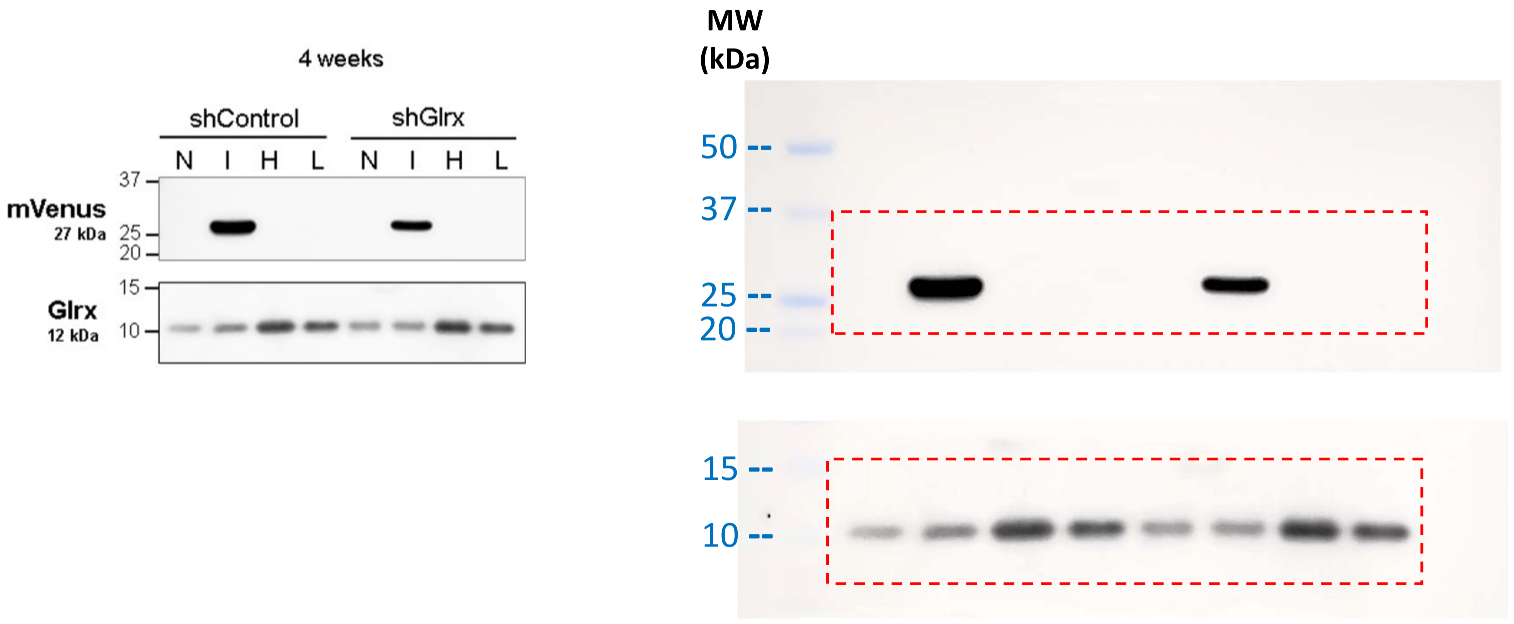

Supplement: Supplementary file 2 — Protocols, Original Blots, and Drawings [file 41598_2019_49624_MOESM2_ESM.pdf]
